# An Overview of Near Infrared Spectroscopy and Its Applications in the Detection of Genetically Modified Organisms

**DOI:** 10.3390/ijms22189940

**Published:** 2021-09-14

**Authors:** Soo-In Sohn, Subramani Pandian, Young-Ju Oh, John-Lewis Zinia Zaukuu, Hyeon-Jung Kang, Tae-Hun Ryu, Woo-Suk Cho, Youn-Sung Cho, Eun-Kyoung Shin, Byoung-Kwan Cho

**Affiliations:** 1 Department of Agricultural Biotechnology, National Institute of Agricultural Sciences, Rural Development Administration, Jeonju 54874, Korea; pandiannsp7@gmail.com (S.P.); happykorean@korea.kr (H.-J.K.); thryu@korea.kr (T.-H.R.); phyto@korea.kr (W.-S.C.); younsung@korea.kr (Y.-S.C.); novis7@korea.kr (E.-K.S.); 2 Institute for Future Environmental Ecology Co., Ltd., Jeonju 54883, Korea; 50joo@hanmail.net; 3 Department of Measurements and Process Control, Szent István University, H-1118 Budapest, Hungary; izaukuu@yahoo.com; 4 Department of Biosystems Machinery Engineering, Chungnam National University, Daejeon 34134, Korea

**Keywords:** chemometric analysis, deep learning, environmental risk, genetically modified organisms, near-infrared, spectroscopy

## Abstract

Near-infrared spectroscopy (NIRS) has become a more popular approach for quantitative and qualitative analysis of feeds, foods and medicine in conjunction with an arsenal of chemometric tools. This was the foundation for the increased importance of NIRS in other fields, like genetics and transgenic monitoring. A considerable number of studies have utilized NIRS for the effective identification and discrimination of plants and foods, especially for the identification of genetically modified crops. Few previous reviews have elaborated on the applications of NIRS in agriculture and food, but there is no comprehensive review that compares the use of NIRS in the detection of genetically modified organisms (GMOs). This is particularly important because, in comparison to previous technologies such as PCR and ELISA, NIRS offers several advantages, such as speed (eliminating time-consuming procedures), non-destructive/non-invasive analysis, and is inexpensive in terms of cost and maintenance. More importantly, this technique has the potential to measure multiple quality components in GMOs with reliable accuracy. In this review, we brief about the fundamentals and versatile applications of NIRS for the effective identification of GMOs in the agricultural and food systems.

## 1. Introduction

**Figure 1:**
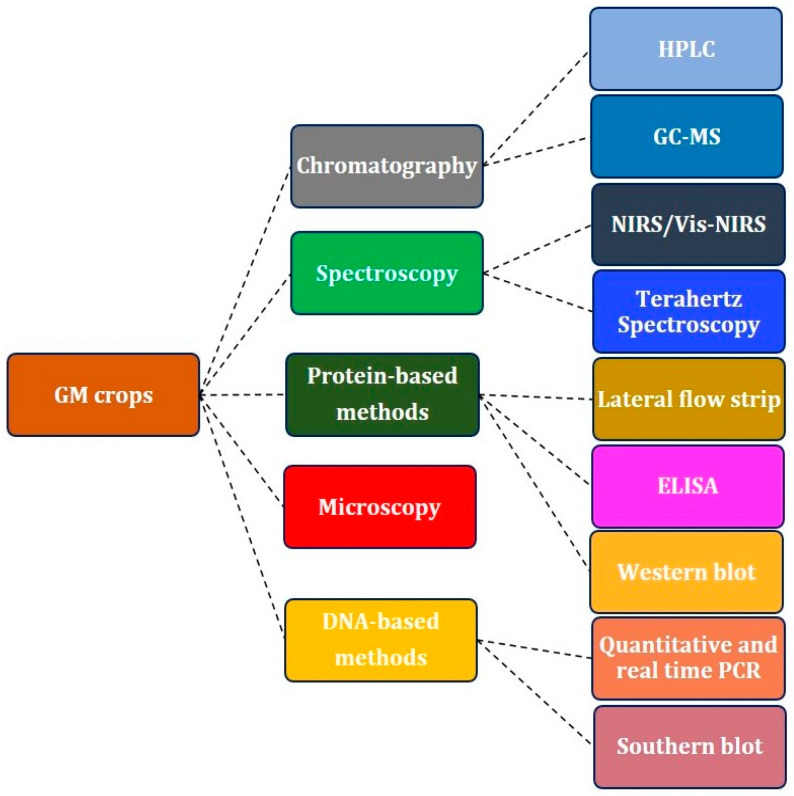
Conventional methods for the identification of GMOs.

Nowadays, genetics has a wide range of applications in various sectors of science, it is used by a variety of techniques and methods, and resulting in a rapid increase in growth rate. The production of genetically modified organisms (GMOs) is one of the most important applications. The number of GMOs is increasing in several countries, particularly in the field of agriculture. Genetically modified (GM) crops have several advantages, such as insect, weed, disease, and drought resistance, improved nutritional value, and increased production [1]. Transgenic plants are grown in 29 countries, and their cultivated area has increased 100 times since 1996, hitting 190.4 million hectares [2,3]. However, across a significant portion of the world, non-governmental organizations and/or the general public are hesitant or opposed to the production and use of GM crops [4]. Furthermore, it has been argued that the use of GM technology could result in unpredicted negative effects on food and environmental safety. In several countries where the commercial cultivation of GM crops is not allowed, GM seeds/products are reportedly, being imported for food and other applications [5]. In this case, unintentional release into the environment is the major issue. For example, it has been reported in various countries, like Japan, Canada, Switzerland and Australia, that GM crops are sometimes found growing on the roadsides as a result of the spilling of GM seeds during transport. Pollen-mediated transfer of uncontrolled foreign genes into nearby wild plants may lead to the production of toxins linked to GM food, and it can change the host plant’s biodiversity by altering the expression of existing genes [6]. Therefore, the regulatory bodies enforce legal pressure to limit the production of GMOs. Consequently, there is a need for precise and inexpensive GMO detection methods. Several DNA and protein based analytical methods, such as polymerase chain reaction (PCR)/restriction enzyme assay and so on (Figure 1), have been used for detection, characterization and authentication of GM crops and their derived agricultural products [7]. Overall, the DNA based methods offer adequate confidence and reliability when compared to other methods for identifying transgenics [8,9]. However, these approaches are destructive, time-consuming, laborious, and expensive, making them unsuitable for online applications [8].

Apart from the DNA/protein-based methods, a few methods like chromatography and spectroscopic techniques such as mid-infrared (MIR), near-infrared (NIR) spectroscopy, terahertz and laser-induced breakdown spectroscopy were found to be effective in the identification of GM crops [10] (Table 1). Among them, near infrared spectroscopy (NIRS) was the most common. NIRS is a rapid and not tedious technology that has been widely used as a non-destructive approach for identifying GMOs. NIRS is a well-known and powerful method for obtaining quantitative data on the chemical and physical characteristics [11] of different biomasses [12]. It includes visible near-infrared (Vis-NIR) and Fourier transformed near-infrared (FT-NIR) spectroscopies. The most significant advantages of this technique over previous GMO research processes are its low cost, little to no sample preparation, and less time-consuming procedures [1]. Another advantage is that there are no chemicals used in the analytical method, making it ecologically friendly. NIRS has been used for varietal identification in various crops, including wheat, maize and rice and for detecting GM crops [13]. It is also being used to detect transgenic foods and adulterations in food products [1,4,14]. The technique could also predict important food components in vital agricultural products such as tomatoes [15] and mung beans [16]. Nonetheless, there is still a major gap with regards to a detailed and thorough application of NIRS from its fundamentals to its application in GMO analysis. In this review, we will focus on the basic principles, test methods, and applications of NIRS for the detection of GMOs.

## 2. Principles and Characteristics of NIRS

NIRS is based on the absorption of light by various materials in the Vis-NIR region of the electromagnetic spectrum. The normal wavelength range for NIR is between 780 and 2500 nm, whereas the spectral range for the Vis-NIR wavelength region range is from 350–2500 nm (Visible (350–780) and NIR (780–2500)) and it overlaps with the optical radiation range (100–1000 nm) [17,18]. The absorptions detected by Vis-NIRS spectroscopy are primarily overtones and combinations of vibrational modes involving C–H, O–H, and N–H chemical bonds [19]. The NIR spectrometers contain a light source, a beam splitter system (wavelength selector), a sample detector, an optical detector, and a data processing/analyzing system (optional). These parts can have varying characteristics and should be chosen based on their intended usage in order to produce an effective and consistent instrument. Most NIRS systems operate either in transmission, reflection, diffuse reflectance, or transflectance mode depending on the type of instrument being used and the type of analysis being performed. Initially, the spectra of samples are collected using an NIR spectrometer. After the collection of data, chemometric analysis is performed to create the calibration model for evaluating the target component (s) using important bands in the NIR spectrum. This step is critical because the precision at this stage ensures that the final calibration model (s) guarantees high reliability. Therefore, the major disadvantage of NIRS is that it always requires reference data for quantitative analysis, which necessitates the use of chemical analysis through conventional analytical instruments [9].

Generally, after collecting the spectra from the spectrometer, the following processes take place: (1) pretreatment or preprocessing of the spectra, (2) building of the calibration models, (3) model transfer, if necessary [20]. There are models and processes for each of the above portions, which are discussed herein. 

### 2.1. Preprocessing Methods

In the first step, generated spectra should be pretreated with specific processes, the main goal of this step is to remove irrelevant information from the collected spectra [21,22]. Besides wavelength selection, baseline correction (Savitzky Golay smoothing filter), multiplicative scatter correction (MSC), noise removal, and scaling are four steps in a typical preprocessing step for NIRS analysis [23]. The objective of the preprocessing procedure might be one of three factors: to enhance a forthcoming exploratory analysis, to improve a subsequent bi-linear calibration model (to compel the data to satisfy Lambert-law), or to improve a subsequent multivariate data analysis model [24]. The two most used preprocessing approaches in NIRS are scatter-correction methods and spectral derivatives. 

**Figure 2:**
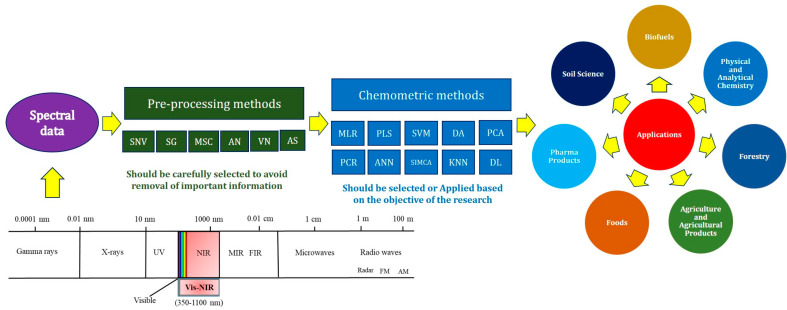
An overview of principle, methods and applications of NIRS.

The scatter-correction methods of preprocessing include Multiplicative Scatter Correction (MSC), Inverse MSC (ISC), Extended MSC (EMSC), Extended Inverse MSC, de-trending, Standard Normal Variate (SNV) and normalization [25]. Furthermore, a wide range of normalization methods, such as mean-centering (MC), auto scaling (AS), vector normalization (VN), and area normalization (AN), are commonly applied in one or more stages of the preprocessing module [22] (Figure 2). 

These methods are intended to decrease the (physical) variability between samples caused by scattering. Martens et al. [26] presented MSC in its basic form, while Geladi et al. [27] further expanded on it. MSC is built on the concept that artifacts or imperfections (for example, unwanted scatter effects) will be excluded from the data matrix prior to data modeling. As Pedersen et al. [28] have pointed out, applying the inversed version of MSC, known as ISC, is quite a simple procedure to apply. The main problem with MSC is defining an appropriate reference spectrum among the multiple spectra [25]. Generally, SNV and normalization methods are based on similar principles. They do not use least squares fitting to estimate their parameters, but they can be susceptible to noisy entries in the spectrum. More robust counterparts of these statistical moments should be used as correction parameters instead of the average and standard deviation methods [29]. Generally, MSC and SNV are two widely known methods that reduce spectral distortions due to scattering. They proved effective in correcting problems of non-homogeneous distribution of the particles and changes in refractive index in food applications [30].

Spectral derivatives have been utilized in analytical spectroscopy for decades because they may eliminate both additive and multiplicative effects in the spectra. The spectral derivative methods include Norris-Williams (NW) derivatives and Savitzky-Golay (SG) polynomial derivatives. All preprocessing methods aim to reduce un-modeled variability in the data to improve the feature sought in the spectra, which is frequently in a linear relationship with a phenomenon (e.g., a constituent) of interest. This can be achieved by using an appropriate preprocessing method, but there is always the risk of employing the incorrect preprocessing technique, which can result in the removal of essential information [25,31]. The NW derivation is a fundamental approach to avoiding noise amplification in finite differences. Norris [32] proposed this methodology and Norris and Williams [33] elaborated on it as a method for calculating the derivative of NIR spectra. The NW derivative works because of the high degree of co-variation and smoothing of the NIR spectra and not necessarily due to spectroscopic reasoning. Savitzky and Golay [34] popularized a method for numerically deriving a vector that includes a smoothing step. The SG is an efficient spectral preprocessing method which has a wide variety of SG modes with a wide scope of applications [25,35]. While simultaneously using various preprocessing methods like SG first derivative, normalization by range, SNV, multiplicative scatter-correction, continuum removed reflectance (CRR), and the transformation to absorbance with different models, it was suggested that CRR could be the best method [36].

### 2.2. Chemometric Analyses

NIR spectra are mainly composed of highly overlapping weak bands. For quantitative analysis of NIR spectra, a multivariate calibration approach should be used. It is becoming more popular as an analytical technique in various fields. The reagent-free NIRS analysis has been established in parallel with chemometric developments, which have high potential for detection of GMOs and various applications. One of its most popular uses is for classification studies using chemometric approaches such as soft independent modeling of class analogies (SIMCA) [37], principal component analysis (PCA) [38], hierarchical cluster analysis (HCA), partial least-squares discriminant analysis (PLSDA) [39], and artificial neural networks (ANNs) [40], linear discriminant analysis (LDA) [41], locally weighted regression (LWR) [42], multivariate adaptive regression splines (MARS) [43], back propagation neural network (BPNN), Moving window partial least squares (MWPLS), least squares-support vector machine (LS-SVM) [44] (Figure 2) and other methods, has been applied to differentiate samples according to the spectral properties [4,45]. These chemometric techniques are often regression-based techniques or classification techniques, and can be either linear or non-linear, supervised or non-supervised methods.

Deep learning is a rapidly emerging field in machine learning that has found widespread use in image and audio recognition [45,46]. Machine learning enables systems to automatically learn and improve based on their experiences. With the emergence of large spectral libraries, we must seize the opportunity to use big data analytics to aid in the use and processing of spectral data, which goes beyond commercial software or packaged machine learning methods [47]. Deep learning-based model is different from traditional neural networks, which have been utilized in NIR spectra processing, as it is made up of multiple processing layers and deeper architectures to learn data representation [45]. Deep learning neural networks may use unprocessed or raw data (such as images or spectra) to automatically find the representations required for prediction. At each layer, the data is modified, magnifying key elements of the input data and suppressing irrelevant data for better prediction [48]. With the emergence of artificial intelligence and deep learning methods, several new model systems, such as Gaussian processes [49], local partial least squares regression [50], convolutional neural networks (CNN) [48], recurrent neural networks (RNNs) [51], fuzzy rule-based systems [52], DeepSpectra model [53], residual neural networks (ResNet), multi-kernel support vector machines [54] have been introduced and have become widely used model systems. Among the most popular deep learning-based models, the DeepSpectra model outperforms all the other model systems [55]. The combination of deep learning with spectroscopic detection methods is a promising approach for quality assessment of food and agro-products and GMO detection [55,56].

## 3. Overview of Biological Applications of NIRS

NIRS has an array of biological applications that include agricultural sciences, agronomy, soil sciences, and so on (Figure 2). Over the past four decades, it has been used to determine the characteristics of agricultural systems, notably in crop and food sciences [56,57]. The NIRS technique is frequently used for variety discrimination [58] and internal properties such as water content, pH, oil content, protein content, fatty acid compositions (oleic acid, erucic acid, etc.), glucosinolate, acid detergent fiber (ADF), sinapate ester content and rigidity in various plant varieties [59,60,61]. The total anthocyanin content of the red-grape homogenates [62], black rice seeds [63] were predicted using NIRS. We can use NIRS for the detection of diseased plants also. Spectral differences between normal and diseased plants can be differentiated using NIRS. Previously, this method was used for identification of disease incidence in plants and postharvest food products [64,65]. Basati et al. [66], have used NIRS for the detection of pest attacks on wheat plants based on pattern recognition as few researchers have detected pesticide residues on the surface of plant leaves and fruits in agricultural fields and forestry [67,68]. 

Although more research on food quality analysis has been conducted, the use of NIRS in food safety evaluation and control is also increasing [69,70]. For example, the quality assessment of lamb meat using NIRS has proven to be an effective technique for assessing tenderness [71], pH, fat, protein, and water content [72], and fatty acid composition in lamb meat [73]. On-line monitoring of meat attributes may also be set up with handheld/portable NIRS, allowing for industrial applications [70,74]. Various applications of NIRS in different food products have been reviewed by several researchers. The in-depth review by Prieto et al. [75] provided an outstanding overview of the ability of NIRS to determine meat chemical composition and quality. In addition, Nicolai et al. [76], Lin and Ying [77] and Chandrasekaran et al. [78] reviewed the use of NIRS to assess the quality and safety of fruits and vegetables. Huang et al. [57] and Wang et al. [79] presented an updated overview of food and beverage quality monitoring. Alishahi et al. [1] and Dale et al. [80] reviewed the use of NIRS to distinguish between transgenic and non-transgenic foods, feeds, and other products. Furthermore, Fu and Ying [81], Qu et al. [69] and Caporaso et al. [82] provided different aspects of the applications of NIRS in food safety measurement and control. Apart from this, NIRS is used as a potential analytical technique in a variety of physical and chemical analyses in various industrial fields [68,83] and also in new emerging fields referred to as aquaphotomics. Aquaphotomics is a new scientific field that is increasingly being explored by many researchers dealing with aqueous systems [16]. It revolves around the principle of using water as a holistic marker to extract information about many different water molecular conformations and their interaction with surrounding solutes by means of their absorbance bands and a light-water phenomenon [84]. Aquaphotomics has been used for noninvasive bio diagnosis and also for measuring low concentrations of sugar in water [84]. 

## 4. Applications of NIRS for the Detection of GM Crops and Transgenic Foods

**Figure 3:**
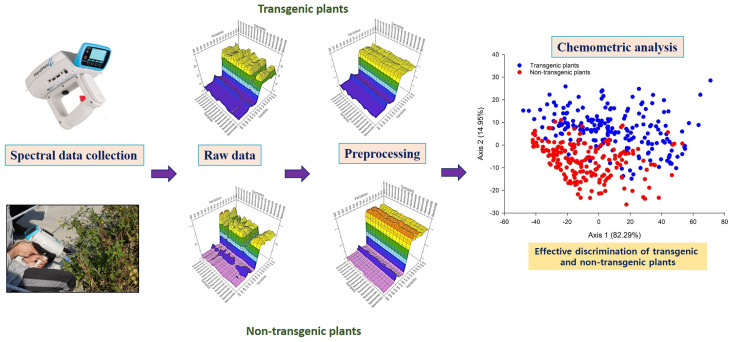
A typical example for application of NIRS with chemometrics on the detection of transgenic plants.

Gene flow from genetically modified organisms might pose a threat to the environment. Hence, it is critical to develop reliable, quick, and low-cost technologies for detecting and monitoring GMOs in crops and their finished products. Researchers have started to explore the potential of NIRS for the rapid detection of GMOs in both laboratory and field conditions (Table 2). A typical example of the evaluation of GM crops using NIRS was shown in Figure 3. Roussel et al. [42] were the first to use NIRS to distinguish Roundup Ready^®^ from conventional soybeans. Roundup Ready^®^ soybean is a glyphosate resistant GM soybean (5-enol-pyruvylshikimate-3-phosphate synthase (*EPSPS* gene) developed by Monsanto that accounts for more than 83% of global annual soybean production [85]. In the study, a database of around 8000 samples yielded an accuracy rate of 93% using PLS, LWR and ANN chemometric models. Concurrently, Munck et al. [86] differentiated normal barley seeds and *lys3a* (high-lysine gene) mutated seeds with both proteomics and NIRS methods. They preprocessed the data with MSC and assessed the spectral data with chemometric analysis (PCA, PLSR) and effectively discriminated the mutant barley seeds with 100% accuracy rate. Later, Rui et al. [87] applied a back-propagation approach to distinguish transgenic maize (*Cry1Ac*) from their parents with 98% accuracy rate by using a continuous wave of NIR diffuse reflectance spectroscopy within the range of 4000–12,000 cm^−1^. Xie et al. [88] also employed Vis-NIRS for the discrimination of transgenic tomatoes with the antisense ethylene receptor (*LeETR2*) gene. For preprocessing, they used MSC and SG 1st and 2nd derivatives, whereas in the case of chemometric analysis, PCA, DA, and PLS-DA were used for effective discrimination with the 100% accuracy rate. In other reports, they have studied tomato plants with antisense *LeETR1* transgene with various chemometric methods like PLS-DA [89], PCA, SIMCA and DPLS [90] with a classification accuracy of 100% (Table 2). Later, they studied the antisense *LeETR2* inserted transgenic tomato with multiple chemometric analyses such as LS-SVM, DA, SIMCA and DPLS (100% accuracy) [91]. In another study, Xie et al. [92], used the SNV method of preprocessing and PCA, DA chemometric analysis for the discrimination of transgenic tomatoes and succeeded with 100% accuracy.

Biradar et al. [93], have used MSC, SNV and SG 1st and 2nd derivatives preprocessing methods for the discrimination of transgenic cotton (*cry1Ac*) from non-transgenic plants. *CrylAc* gene confers resistance to lepidopteron pests. They have used the Vis-NIR spectral range of 400–2500 nm in spectroscopic analysis and by utilizing PLS and PCR chemometric analysis, they have attained a 100% accuracy rate in GM crop detection (Table 2). Jiao et al. [94] used NIR, GC-MS, HPLC, and ICP-AES coupled with chemometric strategies for the discrimination of transgenic rice from non-transgenic rice. Various types of transgenic rice were used in this study, including anti-fungal genes (*RCH10, RAC22,* *β-1,3-Glu* and *B-RIP*), chitinase gene (*RC24*), β-1,3-glucanase gene (*β-1,3-glu*), *p35H* containing a hygromycin phosphotransferase gene (*hpt*) and insect resistant genes (*sck* gene and *cry1Ac*). They have used various preprocessing methods (SVM, SG, first and second derivatives) and different chemometrics (PCA, PLS-DA) for effective discrimination and found a higher level (100% accuracy) of discrimination with PLS-DA chemometric analysis. Lee and Choung [95], evaluated the potential of NIRS in the herbicide resistant transgenic soybean (EPSPS gene) and the non-transgenic soybean. The spectral data from the Vis-NIR region (400–2500 nm) was preprocessed and assessed with chemometric analysis (PCA, PLS-DA) for effective discrimination with the accuracy rate of 100%. Using NIRS, Agelet et al. [96] compared five varieties of Roundup Ready^®^ soybean (*EPSPS* gene) to conventional soybeans. Though the Roundup Ready^®^ has been assessed previously [46], for effective discrimination with advanced chemometric analysis, they have done the experiment and it resulted in successful discrimination with PCA and PLS-DA (100% accuracy rate).

Rice producers face severe economic losses due to insect attacks. Although the use of insecticides can help to mitigate the damage to some extent, it also raises production costs, and pesticide residues lead to serious environmental risks. A better approach appears to be the production of insect-resistant transgenic plants (*cry1Ab, cry1Ac* genes) [97]. The comparative analysis of GMOs using Fourier transformed NIR (FT-NIR), Vis-NIR and MIR spectroscopies for the effective discrimination of GM rice (*cry1Ab*) with its non-transgenic parents was done by Xu et al. [97]. The preprocessing method used was MSV, SNV and SG 1st and 2nd derivatives combined with the chemometric analyses (PCA, DA, PLS-DA) resulted in successful discrimination with the highest accuracy rate (100%). The comparative analysis of multiple spectroscopy methods helps to find the most efficient spectroscopy, preprocessing method, and chemometric analysis for the prediction of transgenic crops. Similarly, Liu et al. [44] used VNIR spectroscopy in combination with chemometric tools (PCA, PLS, PCA-BPNN and LS-SVM) to distinguish GM rice seeds (*cry1Ab/cry1Ac*) from non-GM rice seeds with an accuracy rate of up to 100% using the LS-SVM model (Table 2). Guo et al. [98] also showed that utilizing NIRS detected obvious distinctions between GM and non-GM sugarcane with up to 100% classification accuracy. A total of 456 sugarcane leaf samples, comprising 150 non-transgenic and 306 transgenic with *Bt* and *Bar* genes were studied. They have used the SG and moving-window waveband screening method of preprocessing for the spectra in combination with PCA and LDA analyses for effective discrimination. Long et al. [99] have discriminated between rice lines transformed with a protein gene (*OsTCTP*) and a regulation gene (*Osmi166*) by using NIRS. They used SNV and PLS-DA methods for preprocessing and chemometric analysis respectively and resulted in a 100% classification rate.

NIRS was used by Garcia-Molina et al. [100] to distinguish GM wheat grain and flour from non-GM wheat lines. The RNAi mediated GM wheat with low gliadin (gluten) content was successfully discriminated with the various NIR spectral ranges coupled with chemometric analysis (PLS) with a 99% accuracy rate. Gluten proteins are associated with celiac disease and other complications. Because of their high proline and glutamine concentration, they are referred to as prolamins [100]. Feng et al. [17] have assessed the GM maize (*cry1Ab/cry2Aj-G10evo* proteins) with their non-GM parents by using hyperspectral imaging in the NIR range of 874.41–1733.91 nm combined with chemometric (PCA, SVM and PLS-DA) data analysis. They have discriminated between GM and non-GM maize with a 100% accuracy rate. Feng et al. [18] have assessed the CRIPR-Cas9 mutated rice (*TWG6* gene) and normal rice by using NIRS. The preprocessed (WT) spectra assessed with chemometric methods (SVM, ELM) resulted in higher accuracy (100%) of discrimination (Table 2). Recently, Hao et al. [3] have studied transgenic rice (*cry1Ab/cry1Ac)* for effective discrimination against non-GM rice. In this study, they used multiple preprocessing (NWS, SNV, MSC and SG 1st derivatives) and chemometric analyses (PCA, SVM, PLS-DA) and resulted in 100% accuracy of differentiation capacity.

The NIRS has been used for the discrimination of foods for their quality, longevity, and adulterations were quite common. As compared to transgenic crops, quite a few studies have been performed on the detection of transgenic foods using NIRS. Previously, Zhu et al. [101] used NIRS for the detection of transgenic canola oil in 117 canola oil samples with PCA and DPLS chemometric analyses to assess its feasibility for discrimination. It resulted in a 97.30% accuracy rate for the discrimination of transgenic canola oil by using the DPLS method (Table 2). Later on, Luna et al. [4] used FT-MIR spectroscopy coupled with chemometric analysis (SIMCA, SVM-DA, PLS-DA) for the effective discrimination of transgenic soybean oil and resulted in a 100% accuracy rate. The eighty oil samples were assessed (40 transgenic oil and 40 non-transgenic oil) and the multiple spectral preprocessing methods such as MC, MSC, OSC and SG derivatives (first and second) were used for the discrimination of transgenic oils.

## 5. Conclusions and Future Perspectives

The combination of fundamental science (e.g., plant physiology, biochemistry, and other fields), spectroscopy, and multivariate data analysis enabled the development of technology for reliable and quick on-farm or in-field low-cost analysis. The spectra from NIRS can be used as a fingerprint to elucidate certain compositional features that are difficult to identify using traditional chemical analysis. However, it has a few limitations, such as the low precision and subjectivity of the reference models, which are also barriers to their widespread applications. NIRS sometimes cannot detect or discriminate between molecules/compounds with minor concentrations, but the indirect impacts of such variations can be observed within the spectrum. Thus, more rigorous calibrations are needed in order to improve sampling procedures and reference methods for the commercial applications of NIRS.

**Figure 4:**
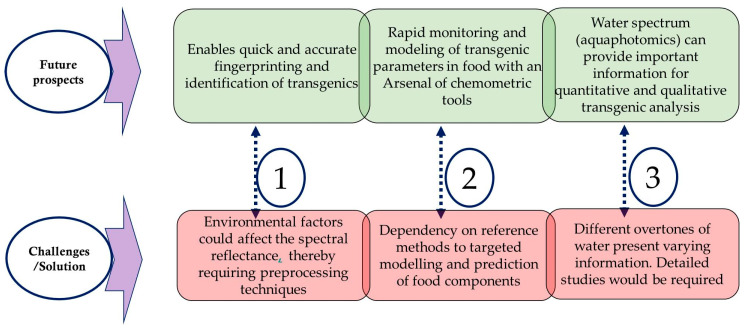
Major future prospects and challenges of NIRS for transgenic food analysis.

In agriculture and food industries, NIRS has become an important analytical technique with multiple applications. Several studies, as discussed above, have demonstrated that NIRS combined with chemometric tools has the potential to discriminate against transgenic crops and foods since it enables quick and accurate identification of transgenics on a larger scale. Although environmental factors could affect the spectral reflectance of the object under the test, the wide availability of preprocessing tools has enabled successful application of NIRS for transgenic analysis with reliable results. Furthermore, studies may be required to develop targeted models for specific component analysis in transgenic foods (Figure 4). Also, studying the spectrum of water through aquaphotomics is a novel field that could be explored for monitoring transgenic foods.

## Figures and Tables

**Table 1:** The different detection methods for genetically modified organisms.

Parameter	Protein-Based			DNA-Based			Microscopy	Chromatography	Spectroscopy	
	Western Blot	ELISA	Lateral Flow Strip	Southern Blot	Qualitative PCR	Real-Time PCR	Classical Microscopy	HPLC and GC-MS	NIRS and Vis-NIRS	TeraHertz Spectroscopy
Ease of use	Difficult	Moderate	Simple	Difficult	Difficult	Difficult	Difficult	Difficult	Simple	Moderate
Needs special equipment	Yes	Yes	No	Yes	Yes	Yes	Yes	Yes	Yes	Yes
Sensitivity	High	High	High	Moderate	Very High	High	High	Very High	High	High
Duration	2 d	30–90 min	10 min	6 h	1.5 d	1 d	1 d	1–2 d	Less than 1 min *	15 min
Cost/sample (US$)	150	5	2	150	250	450	2	20	**	10
Gives quantitative results	No	Yes	No	No	No	Yes	No	Yes	Yes	No
Suitable for field test	No	Yes	Yes	No	No	No	No	No	Yes	In progress
Employed mainly in	Academic labs	Test facility	Field Testing	Academic labs	Test facility	Test facility	Test facility	Test facility	All fields	Test facility
Technical	Yes	Yes	No	Yes	Yes	Yes	Yes	Yes	No	No
Selective	Yes	Yes	Yes	Yes	Yes	Yes	Yes	Yes	No	No
Portable /handheld versions available	No	No	Yes	No	No	No	No	No	Yes	In progress

ELISA, Enzyme linked immunosorbent assay; DNA, Deoxyribonuclic acid; PCR, Polymerase chain reaction; HPLC, High pressure liquid chromatography; GC-MS, Gas chromatography mass spectroscopy; NIR, Near infrared spectroscopy; Vis-NIRS, Visible-Near infrared spectroscopy. *: Depends on instrument type. **: Depends on handheld or benchtop.

**Table 2:** Studies on genetically modified organism detection using near infrared spectroscopy.

S. No	Plant	Gene	Preprocessing Method	Chemometric Analyses	Remarks	Reference
1	Soybean	Roundup Ready^®^ (*EPSPS* gene)	SNV	PLS, LWR, ANN	Around 8000 samples discriminated with an accuracy rate of 93%	[42]
2	Barley	Mutation of *lys3a*	MSC	PCA, PLSR	Effective discrimination of barley seeds	[86]
3	Maize	*cry-gene*	-	-	Effective discrimination with back-propagation approach	[87]
5	Tomato	Antisense *LeETR2*	MSC, SG 1st and 2nd derivatives	PCA, DA, PLS-DA	Effective discrimination of tomato with highest accuracy	[88]
6	Tomato	Antisense *LeETR1*	MSC, SG 1st and 2nd derivatives	PLS-DA	Effective discrimination of tomato with highest accuracy	[89]
7	Tomato	Antisense *LeETR1*	MSC, SG 1st and 2nd derivatives	PCA, SIMCA, DPLS	Effective discrimination of tomato with highest accuracy	[90]
8	Tomato	Antisense *LeETR2*	-	LS-SVM, DA, SIMCA, DPLS	Effective discrimination of tomato with highest accuracy	[91]
9	Tomato	Antisense *LeETR1*	SNV	PCA, DA	Effective discrimination of tomato with highest accuracy	[92]
10	Cotton	*cry1Ac*	MSC, SNV, SG 1st and 2nd derivatives	PLS, PCR	Effective discrimination of cotton with 100% accuracy rate	[93]
11	Rice	*RCH10, RAC22, B-1,3-glu, B-RIP*	SNV, SG 1st and 2nd derivatives	PCA, PLS-DA	A comprehensive study for the GM discrimination with multiple genes and methods	[94]
12	Soybean	*EPSPS* gene	2nd derivatives	PCA, PLS-DA	Using Vis-NIRS for effective discrimination of soybean with 100% accuracy	[95]
13	Soybean	Roundup Ready^®^ (*EPSPS* gene)	-	PCA, PLS-DA	Successful discrimination of Roundup Ready^®^ soybean	[96]
14	Rice	*cry1Ab*	MSC, SNV, SG 1st and 2nd derivatives	PCA, PLS-DA, DA	Comparative analysis using fourier transformed NIR (FT-NIR), Vis-NIR and MIR spectroscopies for the effective discrimination of GM rice	[97]
15	Rice	*Bt cry gene*	CDA	PCA, PLS-DA, LS-SVM, PCA-BPNN	Effective discrimination of GM rice and non-GM rice seeds	[48]
16	Sugarcane	*Bt* and *Bar* genes	SG	PCA, LDA	Effective discrimination of 456 GM and non-GM sugarcane leaf samples	[98]
17	Rice	*OsTCTP* and *Osmi166*	SNV	PLS-DA	Effectively discriminated rice lines transformed with protein (*OsTCTP*) and regulation (*Osmi166*) genes by using NIRS	[99]
18	Bread wheat	RNAi mediated downregulation of gliadin epitopes	SNV/DT	DPLS	Discrimination of RNAi mediated GM wheat with low gliadin (gluten) content	[100]
19	Maize	*cry1Ab/cry2Ag-G10evo*	WT, SNV, MSC	PCA, SVM	Effective discrimination of GM maize lines with 100% accuracy	[18]
20	Rice	CRISPR-Cas9 mediated mutation of *TGW6*	WT	SVM, ELM	Effective discrimination of CRIPR-Cas9 mutated rice (TWG6 gene) and normal rice by using NIRS	[19]
21	Rice	*cry1Ab/cry1Ac*	NWS, SNV, MSC, SG 1st derivatives	PCA, SVM, PLS-DA	Effective discrimination of GM rice lines with highest accuracy rate.	[3]
**Transgenic Foods**						
1	Canola oil	*-*	-	PCA, DPLS	117 canola oil samples were discriminated with a 97.30% accuracy rate	[101]
2	Soybean oil	*-*	Mean centering/MSC	PCA, SVM-DA, PLS-DA	40 transgenic and 40 non-transgenic soybean oil discriminated with 100% accuracy rate	[4]
